# Predictive Markers for the Recurrence of Nonmuscle Invasive Bladder Cancer Treated with Intravesical Therapy

**DOI:** 10.1155/2015/857416

**Published:** 2015-11-23

**Authors:** Yasuyoshi Miyata, Hideki Sakai

**Affiliations:** Department of Urology and Renal Transplantation, Nagasaki University Hospital, 1-7-1 Sakamoto, Nagasaki 852-8501, Japan

## Abstract

High recurrence rate is one representative characteristic of bladder cancer. Intravesical therapy after transurethral resection is often performed in patients with nonmuscle invasive bladder cancer (NMIBC) to prevent recurrence. Bacillus Calmette-Guérin (BCG) and several anticancer/antibiotic agents, such as mitomycin C and epirubicin, are commonly used for this therapy. BCG treatment demonstrates strong anticancer effects. However, it is also characterized by a high frequency of adverse events. On the other hand, although intravesical therapies using other anticancer and antibiotic agents are relatively safe, their anticancer effects are lower than those obtained using BCG. Thus, the appropriate selection of agents for intravesical therapy is important to improve treatment outcomes and maintain the quality of life of patients with NMIBC. In this review, we discuss the predictive value of various histological and molecular markers for recurrence after intravesical therapy in patients with NMIBC.

## 1. Introduction

Urothelial cancer of the bladder is a common disease throughout the world. Approximately two-thirds of all bladder cancers (BCs) are considered nonmuscle invasive bladder cancer (NMIBC) at diagnosis [1]. Transurethral resection (TUR) of the bladder is the standard therapy to remove cancerous tissue from patients with NMIBC. Unfortunately, BC frequently recurs after TUR. In fact, up to 70% of patients with NMIBC experience local BC recurrence after receiving the appropriate treatment. The risk of BC recurrence and progression increases in high grade BC [2]. Intravesical therapy is recommended to reduce the risk of recurrence and progression in these patients. In recent years, various therapeutic agents have been examined in preclinical and clinical trials for use in post-TUR adjuvant intravesical therapy [3, 4]. The most commonly used therapeutic agents include Bacillus Calmette-Guérin (BCG) and a variety of chemotherapeutic agents, even now.

Intravesical immunotherapy with BCG is the most effective therapy to reduce the recurrence and progression of NMIBC and most guidelines recommend BCG therapy for patients with NMIBC [5]. However, intravesical BCG therapy can cause adverse effects, ranging from lower abdominal discomfort and cystitis to bladder atrophy and sepsis. In addition, nearly 40% of patients do not respond to intravesical BCG therapy [6]. A number of patients with NMIBC reject BCG therapy because of the high failure rates and severe adverse effects associated with the therapy. Intravesical therapy with chemotherapeutic or antibiotic agents is another popular therapeutic option. Adverse effects associated with these treatment options are rare and mild. However, some patients in the intermediate- or high-risk groups could be at higher risks of recurrence and progression than if they received intravesical BCG therapy. Therefore, accurate predictions of anticancer effects associated with each intravesical therapy are important when deciding treatment strategies in patients with NMIBC.

In this review, we discuss the prognostic value of various types of predictive factors for recurrence in patients with NMIBC treated with adjuvant intravesical therapy after TUR. Many reports have been published and well-written reviews exist on this topic. However, many of them involve analyses of intravesical BCG therapy. In addition, most of these reviews were published nearly fifteen years ago. Certainly, there are only few new and dramatic topics in this field. However, intravesical therapy with chemotherapeutic agents or BCG continues to be the most commonly used and effective therapy for patients with NMIBC. Therefore, we made special efforts to summarize these treatments based on reports from the past decade. There are many reports on predictive markers in urine samples [7–9]. However, in this review, we discuss the results obtained from tissue samples because intravesical therapy is usually performed after TUR.

The most popular and effective agent for post-TUR intravesical therapy is BCG. Although various types of chemotherapeutic or antibiotic agents are used for this therapy, most studies investigated the anticancer effects of mitomycin C (MMC), epirubicin, and cytarabine (Ara-C). MMC is a chemotherapeutic agent that acts by inhibiting DNA synthesis. Epirubicin is an anthracycline antibiotic agent that demonstrates minimal transurothelial absorption [10]. Ara-C is an antagonist of pyrimidine metabolism that has low incidences of local and/or general adverse effects [11]. Gemcitabine, paclitaxel, and some other newly developed agents have also been investigated in preclinical and clinical trials [3, 4, 12]. We pay special attention to BCG, MMC, and epirubicin in this review because these are currently the most popular agents and most reports regarding predictive markers for cancer recurrence in adjuvant intravesical therapy used these agents.

Numerous studies investigated how to optimize intravesical therapy in patients with NMIBC by examining different durations, timings, and regimens [13]. However, no single best method has been determined. Intravesical therapies vary widely in dosage, duration, and the presence or absence of maintenance therapy even if the same agent was used. Thus, biases and differences in the evaluation of cancer-related and immune-related molecules in human tissues are inevitable. In addition, there is a report that BCG efficacy in Japanese population tended to show decreasing nonrecurrence rates with time whereas nonrecurrence rate in western countries increased each year [14]. Furthermore, in the Asian population, recurrence-free survival between intravesical BCG and intravesical MMC therapy was not significantly different [15]. Therefore, this review is limited by the lack of uniformity in therapeutic policies, evaluation methods, and races analyzed. However, we believe that the information in this review will still be useful for discussing treatment and observation strategies in patients with NMIBC.

## 2. Clinical Background and Pathological Features

Most investigators and urologists initially studied whether certain characteristics of patients (e.g., age, gender, body mass index, and information on adverse effects) could predict clinical outcomes and recurrence after adjuvant intravesical therapy. Most of these studies suggest that these characteristics cannot predict anticancer effects and outcomes after intravesical BCG therapy [21, 30, 31]. Various pathological features and endoscopic tumor characteristics were also analyzed for potential prognostic capabilities in recurrence after intravesical therapy. However, these factors also failed to predict the response and time to recurrence [31]. The expression levels of cancer-related molecules, immune responses, and gene polymorphisms were also analyzed in patients as the next potential prognostic resources. Before discussing the prognosis of BCG therapy in patients with NMIBC, we should note the difference between BCG refractoriness and BCF relapse. Recurrence-free survival and overall survival of BCG-refractory patients are significantly worse compared to those of BCG-relapsing patients [32]. Therefore, further detailed studies with rigid distinction may be essential in the future.

## 3. Cell Proliferation

Cell proliferation is an important regulator of tumor growth, progression, and outcome in patients with NMIBC [33, 34]. Therefore, many studies investigated the relationship between recurrence and cell proliferation indices measured using Ki-67-positive cells (Ki-67 labeling index; Ki-67 LI) [16, 35, 36]. However, a consensus on the value of Ki-67 for predicting recurrence has yet to be reached. For example, patients with a high Ki-67 LI have significantly worse recurrence-free survival after intravesical BCG therapy than those with a low Ki-67 LI [16, 36]. However, while a significant predictive value was detected in a univariate analysis, it was not detected in a multivariate analysis. Another report demonstrated that the Ki-67 LI did not predict recurrence in intravesical BCG therapy [19]. On the other hand, Chen et al. [18] reported that a high Ki-67 LI (>25%) was a significant predictor of recurrence after intravesical therapy with MMC and epirubicin (*P* = 0.001) in 72 patients with pTa or pT1. Ki-67 LI was also identified as an independent prognostic indicator in a multivariate analysis (risk ratio, 2.021; 95% confidence interval, 1.018–4.010; *P* = 0.044). When a similar analysis was performed, high Ki-67 LI (≥20%) was a significant predictive factor for progression-free survival in both univariate and multivariate analyses (*P* = 0.006 and *P* = 0.042, resp.) [17]. However, in this study, Ki-67 LI did not significantly correlate with recurrence even in a univariate analysis [17]. No conclusion can be reached about the prognostic roles of Ki-67 LI for adjuvant MMC and epirubicin therapies because these two studies used different protocols for intravesical therapy, chose different cut-off values for the Ki-67 LI, and examined different pathological features. Thus, Ki-67 LI cannot be considered a useful predictor of recurrence after intravesical therapy. A summary of the predictive values of Ki-67 LI for recurrence after intravesical therapy is shown in Table 1. On the other hand, there was a report regarding proliferating cell nuclear antigen (PCNA) and recurrence in patients with NMIBC treated with intravesical therapy [37]. This study showed that rates for PCNA-positive cancer cells were 52.6% (10/19) in recurrent and 78.9% (15/19) in nonrecurrent cases. Although a significant difference of PCNA was not detected in recurrence, the number of patients was relatively small (*n* = 19).

## 4. Apoptosis and Apoptosis-Related Molecules

The induction of cell death by apoptosis is an important anticancer effect of intravesical therapy [38]. Therefore, semiquantification of apoptosis may be a useful predictive marker of recurrence after this therapy. The most popular methods of semiquantification of apoptosis in tissue samples are currently TdT-mediated dUTP nick end labeling (TUNEL) and the quantification of caspase-3 levels [39, 40]. These methods were also used in many studies investigating the relationships between apoptosis and carcinogenesis, pathological features, and survival in patients with diseases, including BC [39–41]. However, to our knowledge, only few reports are investigating the prognostic role of the apoptotic index measured by TUNEL and/or caspase-3 expression after intravesical therapy in patients with NMIBC. However, several apoptosis-related molecules were investigated as potential predictive tools and are summarized in Table 1.

### 4.1. TP53

Wild type p53 is a well-known regulator of cell cycle control in normal cells and acts as a tumor suppressor by controlling apoptotic processes under genotoxic conditions. However, mutations in the p53 gene can induce cell cycle deregulation and apoptosis in cancer cells. Nuclear accumulation of p53 influences the malignant potential and aggressiveness of NMIBC [32, 42]. The prognostic abilities of p53 expression during recurrence after intravesical BCG therapy were investigated, but conflicting results were obtained [17, 19–22, 36, 37, 42–45]. However, as shown in Table 1, the current opinion is that p53 expression has minimal value in predicting recurrence after intravesical BCG therapy. On the other hand, a different study found a mutation in the gene encoding p53 that is associated with the recurrence after intravesical BCG therapy [46]. However, this result was achieved by a univariate analysis and the study included a relatively small sample size (*n* = 26). Thus, although the pathological significance and prognostic role of p53 have been widely investigated, the results of these studies are not conclusive enough to make a clinical decision. On the other hand, in recent years, a meta-analysis demonstrated that p53 overexpression was associated with recurrence-free survival in patients with NMIBC treated with intravesical BCG therapy in the Asian population (hazard ratio = 1.57, 95% confidential interval = 1.08–2.27) [47]. This meta-analysis has several limitations, including presence of heterogeneity, publication bias, and a wide range of cut-off points. However, we support the opinion that there is value in continuing further studies with rigid criteria and large population.

### 4.2. p63

The p63 protein is a member of the p53 tumor suppressor protein family [48]. Levels of p63 expression in NMIBC were reported to be higher than those in MIBC [49]. ΔNp63 is an isoform of p63 and it was also reported to act as a tumor suppressor in various cancers [48, 50]. The prognostic potential of p63 and ΔNp63 expression levels was recently investigated in 134 patients with pT1 high-grade cancer (131 of 134 patients (97.8%) that were treated with adjuvant intravesical BCG therapy) [22]. They found that ΔNp63 expression was a significant predictive factor in both univariate and multivariate analyses, whereas p63 expression was not (Table 1). Unfortunately, the molecular mechanisms underlying the prognostic capabilities of ΔNp63 expression are unclear.

### 4.3. Survivin

Survivin is a member of the inhibitor of apoptosis (IAP) family and it controls various pathological activities by regulating mitosis- and apoptosis-related factors [51]. In patients with BC, survivin expression was associated with malignant potential and recurrence [23, 52]. Univariate and multivariate analyses also demonstrated that survivin expression was a significant and independent predictor of recurrence in 74 patients with NMIBC (*P* = 0.003 and *P* = 0.02, resp.) [23]. Among these 74 patients, however, intravesical therapy was performed in only 54 (73.0%) and the therapies used different agents (BCG or MMC). Although further studies are necessary to conclude the prognostic ability of survivin expression in patients with NMIBC treated with intravesical therapy, this finding facilitates a discussion of the different treatment strategies using intravesical therapy. On the other hand, heterozygous genotypes (GC) of survivin (31G>C) are significantly correlated (*P* = 0.0009) with the recurrence in patients with NMIBC treated with intravesical BCG therapy [24]. These authors also found a considerable relationship with the homozygous genotype (CC) of survivin (31G>C) [24].

### 4.4. Bcl-2 Family

The apoptotic-related molecules Bax and Bcl-2 are strong pro- and antiapoptotic molecules, respectively. Some studies suggest that these molecules contribute to disease aggressiveness and outcome in BC. However, the opposite results have also been found [32, 37, 53]. A higher Bcl-2/Bax ratio (>1) is associated with early recurrence after adjuvant intravesical chemotherapy [53]. A different report found that Bax expression is significantly associated with the risk of recurrence in intravesical BCG therapy, as determined by univariate analysis (*P* = 0.034) [54]. This study also showed that a model, including pT1 stage, age, and expression levels of Bax and Bcl-2, was the best independent and significant predictor for recurrence after therapy. However, we should note that the study population was relatively small (*n* = 28). As the authors noted, further investigations on a larger cohort are necessary to confidently determine the prognostic roles of Bcl-2 and Bax for intravesical BCG therapy in patients with NMIBC.

## 5. Angiogenesis and Angiogenesis-Related Molecules

Angiogenesis is a crucial step for tumor growth and progression in almost all types of cancers, including BC [55–58]. In cancer tissues, microvessel density (MVD) is commonly used to evaluate the angiogenic status. Several investigators demonstrated that MVD was significantly associated with the prognosis and recurrence in BC patients [55, 57, 58]. However, the relationship between MVD and recurrence in patients with NMIBC treated with intravesical therapy contradicted previous expectations. MVD was found to be closely associated with recurrence after intravesical BCG therapy as determined by both univariate (*P* < 0.0001, Table 1) and multivariate analyses (hazard ratio, 4.35; 95% confidence interval, 0.90–21.18; *P* = 0.0011) [25]. However, this study population was relatively small (*n* = 26) and the 95% confidence lower limit value was under 1.0. On the other hand, vascular endothelial growth factor (VEGF) promotes angiogenesis in various physiological and pathological conditions. One report found that increased VEGF immunoreactivity is a worse predictor of recurrence in patients with NMIBC treated with intravesical MMC or epirubicin therapy [18]. As for MVD, there is little information regarding the prognostic value of VEGF after intravesical therapy.

## 6. Other Cancer-Related Molecules

The retinoblastoma protein (pRB) is a well-known tumor suppressor that acts by regulating the cell cycle. One report indicated that pRb expression was not associated with recurrence after intravesical BCG and interferon-alpha therapy (*P* = 0.047) [45]. On the other hand, Cormio et al. [21] reported that altered pRB expression was significantly associated with disease-free survival (*P* = 0.037) in a Kaplan-Meier survival analysis. They also investigated the predicative value of a combined pRb and p53 marker in these patients, but this marker was not significantly associated with survival (*P* = 0.08).

E2F4 is a member of the E2F transcription factor family. E2F4 is involved in cell cycle regulation and the suppression of tumor growth. This protein can bind pRB and there is a report that E2F4 expression level is a useful predictive marker for the effectiveness of intravesical BCG therapy that can predict clinical outcomes, including recurrence, progression, and survival in patients with BC [26]. However, detailed pathological significance and prognostic roles of E2F4 after intravesical therapy are still largely unknown.

Ezrin expression levels are reportedly correlated with cell survival, migration, and adhesion in several cancers [59, 60]. Lower expression levels (<20%) of ezrin in cell membranes are significantly associated with poor disease outcomes in patients with T1 grade 3 disease after intravesical BCG therapy (*P* = 0.041) [34]. Ezrin is a member of the ezrin, radixin, and moesin (ERM) cytoskeleton-associated protein family. This family is composed of ezrin, radixin, and moesin. Currently, the prognostic roles of radixin and moesin in patients with NMIBC are still unknown.

Mutations in fibroblast growth factor receptor- (FGFR-) 3 have been reported to play important roles in disease progression and outcome in patients with certain malignancies, including BC [61]. However, some reports demonstrated a lack of significant relationship between FGFR-3 mutations and disease recurrence in BC [62, 63]. In addition, Park et al. [19] reported that altered FGFR-3 expression was not associated with recurrence after intravesical BCG therapy.

Phosphatase and tensin homolog (PTEN) plays important roles in tumor suppression via the inhibition of the Akt pathway, and its expression was reported to be decreased in NMIBC [19, 64]. One study investigated the ability of PTEN expression levels to predict recurrence after intravesical therapy in T1 grade 3 tumors, but no significant association was detected [19].

CD9, a motility-related protein, is a member of the transmembrane-4 superfamily. The ability of CD9 to inhibit cell motility has been reported in several cancers. However, there is limited information regarding the pathological roles of CD9 in BC [65]. To our knowledge, only one study has investigated the ability of CD9 expression levels to predict recurrence after intravesical BCG therapy, but no significant association was found (*P* = 0.306) [19].

## 7. Immune System

The precise mechanisms of the antitumor effects of BCG in the urinary bladder are not fully understood. Many investigators believe that the response to intravesical BCG therapy depends on a patient's ability to generate adequate, massive, and complex immune responses. In addition, a report shows that BCG strains are associated with the clinical impact in NMIBC; 5-year recurrence-free survival rates of patients treated with BCG Connaught are better than those of patients treated with BCG Tice (*P* = 0.0108) [66]. However, other investigators showed no significant difference (*P* = 0.896) between BCG Tokyo and Connaught strains in the 2-year recurrence-free survival rate [67]. On the other hand, regarding immunization against mycobacterial antigens, the maximal peripheral immune response is observed after 4 weekly BCG instillations in patients previously immunized. However, patients not previously immunized required 6 weekly instillations to achieve a maximum stimulation level [68]. Thus, anticancer effects of BCG are regulated by complex immune-related mechanisms.

BCG antigen presentation leads to the regulation of various types of immune cells (e.g., T lymphocytes, natural killer (NK) cells, NKT cells, and macrophages) and changes in the immunologic tumor microenvironment [30, 69]. Anticancer and antibiotic agents can directly affect the immune system by stimulating inflammation. Therefore, in this chapter, we summarize the predictive value of immune-related factors in patients with NMIBC treated with intravesical therapy (Table 2).

### 7.1. Immunologic Tumor Microenvironment

Nunez-Nateras et al. [30] investigated the relationship between the response to intravesical BCG therapy and pretreatment of the immunologic tumor microenvironment. They investigated the prognostic roles of eosinophil infiltration (Emax) and degranulation (Edgn) and the eosinophil activity index (EAI), which is calculated as Emax + Edgn. They also investigated the prognostic capabilities of the GATA-3^+^ Th2-polarized to T-bet^+^ Th1-polarized lymphocyte (G/T) ratio and the Th2 signature biomarker (Th2 SB), which is calculated as Emax + Edgn + G/T. When these markers were compared between patients that responded to BCG and patients that did not respond to BCG, all markers in responding patients were considerably higher than those in nonresponding patients (Table 2). These authors suggest that the evaluation of Th1 versus Th2 polarization prior to intravesical BCG therapy is useful to predict the response to this therapy. This information is interesting and important to advance our understanding of treatment strategies. However, more detailed and larger studies are necessary to make confident conclusions, because this study had a relatively small (*n* = 38) and nonrandom study population (pTis only).

Another report investigated the relationship between the antitumor effects of intravesical BCG therapy and the expression of antigen-presenting molecules and chemokines [27]. This report showed that patients that did not experience recurrence had increased expression levels of antipresenting molecules and chemokines such as CD1a, CD1b, CD1c, CD1e, major histocompatibility complex- (MHC-) 1, monokine induced by *γ*-interferon (MIG), and interferon-inducible protein 10 kDa (IP-10), after intravesical BCG therapy.

### 7.2. Tumor-Associated Macrophage (TAM)

The immune response has an impact on the anticancer effects of intravesical BCG therapy. Therefore, many investigators have paid special attention to the prognostic roles of immune cells in BC. Tumor-associated macrophages (TAMs) are associated with malignant aggressiveness and prognosis in various types of cancers, including BC [70, 71]. Several reports have shown that TAM density can predict recurrence in patients with NMIBC [29, 72, 73]. For example, in intravesical BCG therapy, recurrence-free survival in patients with lower numbers of TAMs was better than that of patients with higher TAM counts (*P* = 0.0002) [29]. On the other hand, macrophages exist in two different polarization states classified as M1 and M2. M1 macrophages demonstrate anticancer abilities by promoting various tumor-killing mechanisms, while M2 macrophages suppress cancer-related immune responses, stimulating tumor development and progression [72, 73]. Thus, TAMs in cancer tissues have opposite functions against tumorigenesis and immune activity. Suriano et al. [74] investigated the predictive value of total TAMs as well as M1 and M2 macrophage infiltration after intravesical BCG therapy. Similarly to previous reports [72, 73], they found that total macrophage infiltration is a significant predictor of disease-free survival (*P* = 0.020). In addition, Kaplan-Meier survival analysis showed that a low density of M1-TAM and a high density of M2-TAM were significantly worse predictors of disease-free survival compared to a high density of M1-TAM and a low density of M2-TAM (*P* = 0.029 and *P* = 0.02, resp.) in patients with NMIBC treated with intravesical BCG therapy [74]. These results are interesting and provide critical information about the prognostic roles of TAMs in intravesical BCG therapy. However, the independent roles of M1- and M2-TAMs were not supported by a multivariate analysis.

### 7.3. Other Immune Cells and Immunity-Related Factors

One study indicated that tumor infiltrating dendritic cell (TIDC) levels, evaluated by CD83 expression, were not associated with recurrence in patients treated with intravesical BCG therapy (*P* = 0.210) [28]. However, when a similar analysis was performed in patients treated with more than one maintenance BCG cycle, TIDC levels were positively associated with the risk of recurrence in both univariate and multivariate analyses (*P* = 0.045 and *P* = 0.039, resp.) [28].

Yutkin et al. [75] investigated the predictive value of NK cell receptor ligands for recurrence after intravesical BCG therapy. Interactions between NK cells and their targets influence natural cytotoxicity receptors under pathological conditions, including cancer. Three natural cytotoxicity receptors (NKp30, NKp44, and NKp46) are currently known. The authors investigated the prognostic roles of these three receptors after intravesical BCG therapy. High expression levels of NKp30, NKp44, and NKp46 were significantly associated with favorable treatment responses (*P* = 0.0026, *P* = 0.0027, and *P* = 0.044, resp.) [75]. However, these results were obtained by univariate analyses only and included a relatively small number of patients (*n* = 17). However, we believe that these findings are critical to understand the immune mechanisms responsible for preventing recurrence in patients with NMIBC treated with intravesical BCG therapy.

In recent years, cytokine panel for response to intravesical therapy (CyPRIT) constructed using urinary levels of inducible cytokines was reported as a potential useful predictor for the risk of recurrence during intravesical BCG therapy [76]. Thus, we agree with the opinion that more detailed and wider analyses of cytokines may lead to the identification of useful predictive factors to guide modifications of the dose and duration of BCG immunotherapy in patients with NMIBC [77].

## 8. Gene Polymorphism

Polymorphisms of several genes are associated with macrophage susceptibility to intracellular mycobacterial growth, tuberculosis infection, and response to BCG infection [78–80].* NRAMP1* is one of these macrophage susceptibility-related genes. Several studies investigated the relationships between* NRAMP1* gene polymorphisms and disease outcomes in patients with NMIBC treated with intravesical BCG therapy [81, 82]. Of 22 tumors with the* NRAMP1* D543N G:A genotype, cancer recurred in 4 patients (18.2%). The median time to recurrence was 104.6 months. Of 47 tumors with the* NRAMP1* D543N G:G genotype, cancer recurred in 19 patients (40.4%) and the median time to recurrence was 80.2 months. Thus, the* NRAMP1* D543N G:G genotype was associated with a higher frequency of recurrence and a shorter time to recurrence in patients treated with intravesical BCG therapy (*P* = 0.033). This genotype is recognized as an independent and significant predictive factor for the time to recurrence after intravesical BCG therapy (hazard ratio, 4.57; 95% confidence interval, 1.367–15.272; *P* = 0.014). This study showed that the* NRAMP1* (GT)n allele 3 was also associated with decreased recurrence time in a similar multivariate analysis model (hazard ratio, 24.789; 95% confidence interval, 3.074–199.883; *P* = 0.03).

The same study also showed that the* hGPX1* CT genotype (Pro-Lue) predicts shorter recurrence times after intravesical BCG therapy (*P* = 0.03) [80]. hGPX1 is a selenium-dependent enzyme and it participates in the detoxification of hydrogen peroxide and oxide radicals. The codon 198 variant of* hGPX1* is reportedly associated with tumor development [83]. A significant relationship between* hGPX* polymorphism and recurrence in patients with NMIBC has also been previously reported [84]. However, this study population included 128 patients treated with intravesical therapy by BCG and 96 patients treated with other agents. Although further studies are necessary to confidently assess the predictive value of* hGPX* polymorphism for outcome after intravesical BCG therapy, we believe that this gene is a likely candidate prognosis marker. In recent years, relationships between genetic variation in glutathione (GSH) pathways and recurrence in patients with NMIBC treated with BCG after TUR were analyzed. This study showed that 7265992 in GSH synthetase was the most significant (hazard ratio = 3.43, 95% confidential interval = 2.19–13.46, *P* = 0.0003) single nucleotide polymorphism in these patients [85].

## 9. Concluding Remarks

Intravesical therapy is used to prevent recurrence in patients with NMIBC. However, intravesical BCG therapy commonly causes adverse effects. Intravesical therapy with other anticancer agents or antibiotics are relatively safe, but they present lower anticancer effects than BCG. Therefore, predictive markers for anticancer effects, including recurrence prevention, are important tools when selecting treatment strategies for these patients. However, our current understanding of the predictive markers for recurrence after intravesical therapy is insufficient. Despite the fact that many studies have been performed, few results have been verified by multivariate analyses and there is no predictive marker in clinical use. Treatment strategies by intravesical approaches, based on new concepts and ideas, have been recently proposed. Further* in vivo* and* in vitro* studies investigating prognostic markers capable of predicting anticancer effects, clinical outcomes, and adverse effects in new intravesical therapies are required.

## Figures and Tables

**Table 1:** Predictive value for recurrence in cancer-related factors and molecules.

Variable	Patients		Agent	*P* value for recurrence		Year/reference
	*N*	Background		Univariate	Multivariate	
Ki-67	92	Tis, T1G3	BCG	0.015		2009/[16]
	129	T1G3	MMC	0.517		2010/[17]
	72	Ta, T1	MMC/epirubicin	0.011	0.044	2012/[18]
	61	T1G3	BCG	0.677		2013/[19]

P53	53	Tis, Ta, T1	BCG	0.741		2007/[20]
	27	T1G3	BCG	0.92		2009/[21]
	129	T1G3	MMC	0.452		2010/[17]
	61	T1G3	BCG	0.794		2013/[19]
	134^*∗*^	T1 HG	BCG	0.830		2015/[22]

pRb	53	Tis, Ta, T1	BCG	0.580		2007/[20]
	27	T1G3	BCG	0.037		2009/[21]
	61	T1G3	BCG	0.951		2013/[19]

Survivin	74^*∗∗*^	Tis, Ta, T1	BCG/MMC	0.003	0.02	2007/[23]
	78	Ta and T1	BCG	0.009		2012/[24]
	78	Ta and T1	BCG	0.043		2012/[24]

p27	61	T1G3	BCG	0.822		2013/[19]

p63	134^*∗*^	T1 HG	BCG	0.129		2015/[22]
ΔNp63	134^*∗*^	T1 HG	BCG	<0.001	*∗∗∗*	2015/[22]

PTEN	61	T1G3	BCG	0.306		2013/[19]

FGFR3	61	T1G3	BCG	0.355		2013/[19]

Ezrin	92	Tis, T1G3	BCG	0.041		2009/[16]

CD9	61	T1G3	BCG	0.760		2013/[19]

MVD	26	Ta, T1	BCG	<0.0001	0.0011	2011/[25]

AT1R	26	Ta, T1	BCG	<0.0001	0.0012	2011/[25]

VEGF	72	Ta, T1	MMC/epirubicin	0.010	0.036	2015/[26]

BCG: Bacillus Calmette-Guérin; MMC: mitomycin C; Rb: retinoblastoma; PTEN: phosphatase and tensin homolog; FGFR: fibroblast growth factor receptor; MVD: microvessel density; AT1R: Angiotensin II Type I Receptor.

^*∗*^One hundred thirty-one (97.8%) patients were treated with adjuvant intravesical BCG therapy.

^*∗∗*^ Among 74 patients, 54 (73.0%) were treated with intravesical BCG or MMC therapy.

^*∗∗∗*^Hazard ratio and 95% confidential interval were 0.383 and 0.193–0.961, respectively. *P* value was not shown.

**Table 2:** Predictive value of immunologic microenvironment and immune cells.

Variable	Patients		Agents	*P* value for recurrence		Year/reference
	*N*	Background		Univariate	Multivariate	
CD1a	59	NMIBC	BCG	0.005		2009/[27]
CD1b	59	NMIBC	BCG	<0.000		2009/[27]
CD1c	59	NMIBC	BCG	0.03		2009/[27]
CD1e	59	NMIBC	BCG	0.007		2009/[27]
MHC-1	59	NMIBC	BCG	<0.000		2009/[27]
MIG	59	NMIBC	BCG	<0.0001		2009/[27]
IP10	59	NMIBC	BCG	<0.0001		2009/[27]

TAM	66	NMIBC	BCG	0.101	0.013	2009/[28]
CD68	41	Tis	BCG	0.0002		2009/[29]

TIDC	66	NMIBC	BCG	0.210		2009/[28]
CD83	30	NMIBC	BCG^*∗*^	0.045	0.039	2009/[28]

Emax	38	Tis	BCG	0.01		2014/[30]
Edgn	38	Tis	BCG	0.04		2014/[30]
EAI	38	Tis	BCG	<0.004		2014/[30]
G/T ratio	38	Tis	BCG	<0.001		2014/[30]
Th2 SB	38	Tis	BCG	<0.0001		2014/[30]

BCG: Bacillus Calmette-Guérin; MHC: major histocompatibility complex; MIG: monokine induced by *γ*-interferon; IP10: interferon-inducible protein 10 kDa; Emax: eosinophil infiltration; TAM: tumor-associated macrophage; TIDC: tumor infiltrating dendritic cells; Edgn: eosinophil degranulation; EAI: eosinophil activity index; G/T: GATA-3^+^ lymphocytes/T-Bet^+^ lymphocytes; Th2 SB: Th2 signature biomarker.

^*∗*^Patients treated with more than one maintenance BCG cycle.
